# Mapping of quantitative trait locus reveals *PsXI* gene encoding xylanase inhibitor as the candidate gene for bruchid (*Callosobruchus* spp.) resistance in pea (*Pisum sativum* L.)

**DOI:** 10.3389/fpls.2023.1057577

**Published:** 2023-01-30

**Authors:** Jianjun Yan, Jingbin Chen, Yun Lin, Xingxing Yuan, Prakit Somta, Yaowen Zhang, Zeyan Zhang, Xianhong Zhang, Xin Chen

**Affiliations:** ^1^ College of Plant Protection, Shanxi Agricultural University, Shanxi, China; ^2^ College of Agriculture, Shanxi Agricultural University, Shanxi, China; ^3^ Institute of Industrial Crops, Jiangsu Academy of Agricultural Sciences, Nanjing, China; ^4^ Department of Agronomy, Faculty of Agriculture at Kamphaeng Saen, Kasetsart University, Nakhon Pathom, Thailand

**Keywords:** pea, *Pisum sativum*, bruchid, *Callosobruchus*, QTL, xylanase inhibitor

## Abstract

Pea (*Pisum sativum* L.) is an important legume crop for both food and feed. Bruchids (*Callosobruchus* spp.) are destructive insect pests of pea in the field and during storage. In this study, we identified a major quantitative trait locus (QTL) controlling seed resistance to *C. chinensis* (L.) and *C. maculatus* (Fab.) in field pea using F_2_ populations derived from a cross between PWY19 (resistant) and PHM22 (susceptible). QTL analysis in the two F_2_ populations grown in different environments consistently identified a single major QTL, *qPsBr2.1*, controlling the resistance to both bruchid species. *qPsBr2.1* was mapped onto linkage group 2 between DNA markers 18339 and PSSR202109 and explained 50.91% to 70.94% of the variation in resistance, depending on the environment and bruchid species. Fine mapping narrowed down *qPsBr2.1* to a genomic region of 1.07 Mb on chromosome 2 (chr2LG1). Seven annotated genes were found in this region, including *Psat2g026280* (designated as *PsXI*), which encodes a xylanase inhibitor and was considered as a candidate gene for bruchid resistance. PCR amplification and sequence analysis of *PsXI* suggested the presence of an insertion of unknown length in an intron of PWY19, which causes variation in the open reading frame (ORF) of *PsXI*. Moreover, the subcellular localization of *PsXI* differed between PWY19 and PHM22. These results together suggested that *PsXI* encoding xylanase inhibitor is responsible for the bruchid resistance of the field pea PWY19.

## Introduction

Pea (*Pisum sativum* L.) is an important cool season legume crop (Teshome et al., 2015). It is a temperate legume that is widely cultivated globally, with Canada, Russia, China, and India being the major pea-producing countries (FAO, 2020). Seeds of pea are rich in protein (24.0%–32.4%), starch (45.5%–54.2%), and minerals (Bastianelli et al., 1998; Gabriel et al., 2008; Smýkal et al., 2012). In addition, young leaves of pea are high in proteins, minerals, and vitamins, and are edible. Thus, pea serves as a major source of nutrients for humans and livestock (Teshome et al., 2015).

A major problem facing field pea production is seed damage caused by bruchid beetles (bruchids). Bruchids or seed weevils (Coleoptera: Bruchidae) are stored product insects that ingest starchy seeds of legumes and cereal crops (Southgate, 1979). Pea weevil (*Bruchus pisorum* L.), azuki bean weevil (*Callosobruchus chinensis* L.), and cowpea weevil (*Callosobruchus maculatus* F.) are among the most serious and widespread bruchid beetles infesting pea during storage (Hardie et al., 1995; Umrao and Verma, 2002; Duan et al., 2014; Sharma et al., 2016; Aznar-Fernández et al., 2017). The bruchids initially infest the pods and seeds of legumes in the field. Secondary infestation during storage, in which the bruchid population grows rapidly, is much more serious and often results in complete loss of seed lots within 3–4 months (Banto and Sanchez, 1972). Legume seeds infested by bruchids are unfit for human and animal consumption, agricultural use, and trading (Srinives et al., 2007; Deshpande et al., 2011; Yadav, 2018). Although several methods such as chemical control and physical control have been used to control bruchids, these methods are managerially and economically impractical to smallholders and/or hazardous to farmers, consumers, and the environment. Instead, the use of bruchid-resistant cultivars is the most efficient and economical way of controlling bruchids (Somta et al., 2007).

Several sources of resistance to bruchids have been identified in peas. The screening of 1,900 *Pisum* accessions for field resistance to *B. pisorum* at nine sites over 4 years revealed 21 accessions with high resistance or immunity, comprising 11 *P. sativum* (cultivated pea) accessions and 10 *P. fulvum* (wild pea) accessions (Hardie et al., 1995). Greenhouse screening of 29 *P. fulvum* accessions from various origins against *B. pisorum* also showed that several accessions were highly resistant and such resistance was due to seed antibiosis (Hardie et al., 1995; Clement et al., 2002). Screening of 100 *P. sativum* accessions from China to *C. chinensis* under laboratory conditions identified two cultivars, ‘Woyaowandou’ and ‘Macaiwandou,’ that are immune to *C. chinensis* (Duan et al., 2014). In addition, field and greenhouse screening of 602 *P. sativum* accessions from Ethiopia to *B. pisorum* revealed four accessions, ‘32454,’ ‘235002,’ ‘226037,’ and ‘32410,’ showing moderate resistance (Teshome et al., 2015). Moreover, field screening of 52 pea accessions to *B. pisorum* in several different environments demonstrated that accessions ‘P669’ (*P. sativum* ssp*. elatis*) and ‘P656’ (*P. fulvum*) showed low rates of damaged seeds, while accessions ‘P314’ (*P. sativum* ssp*. elatis*) and ‘P1’ (*P. abyssinicum*) showed prolonged bruchid development, and accession ‘P665’ (*P. sativum* ssp. *syriacum*) showed resistance at both pod and seed levels (Aznar-Fernández et al., 2017). Furthermore, in screening of the resistance of only seven pea accessions to *C. chinensis*, ‘AWP 600’ (*P. fulvum*), ‘AWP 601’ (*P. fulvum*), ‘AWP 442’ (*P. elatis*; wild pea), and ‘ACP 11’ (cultivated pea) were found to be immune to this bruchid. Nonetheless, there are only a few reports on the genetics and breeding of bruchid resistance in pea. For example, Byrne et al. (2008) reported that seed resistance to *B. pisorum* in the *P. fulvum* accession ATC113, a resistant accession reported by Hardie et al. (1995), is controlled by three recessive genes, *pwr1*, *pwr2*, and *pwr3*, with additive effects and dominant epistasis towards susceptibility. In addition, quantitative trait loci (QTLs) controlling resistance to *B. pisorum* were reported for ATC113 (Aryamanesh et al., 2014) and wild pea *P. sativum* ssp. *syriacum* accession P665 (Aznar-Fernández et al., 2020).

To the best of our knowledge, no reports have yet been published on the genetics and genomics of resistance to *C. chinensis* and *C. maculatus* in pea. *C. chinensis* and *C. maculatus* are Old World bruchid species that have become cosmopolitan bruchid pests of grain legumes due to seed trade (Srinives et al., 2007). These bruchid species have wide legume host ranges (Southgate, 1979). In China, the country with the highest pea production globally (FAO, 2020), *C. chinensis* and *C. maculatus* are the most economically damaging important bruchids, which cause serious seed losses of field pea. Breeding of field peas that are resistant to these insects is an important goal in pea cultivar development. A major limitation of breeding for bruchid resistance is the difficulty of evaluating such resistance, which is time-consuming and resource-intensive. Marker-assisted selection (MAS) has been proven to accelerate the development of bruchid-resistant cultivars through backcrossing by providing rapid, efficient, and precise selection of plants possessing resistance genes/alleles, aided by MAS’s ability to avoid the need to evaluate resistance (Wu et al., 2022).

Previously, we screened accessions of 70 cultivated field peas for resistance to *C. chinensis* and *C. maculatus* and found that PWY19 showed moderate resistance to these bruchid species. PWY19 was received as ‘Woyaowandou,’ a landrace from China, which has been reported to be resistant to *C. chinensis* (Duan et al., 2014). PWY19 was tested for resistance to *C. chinensis* and *C. maculatus* in Thailand, with the results showing that it was moderately resistant to bruchids (P. Somta, unpublished data). Therefore, the objective of this study was to identify QTLs and candidate genes controlling resistance to *C. chinensis* and *C. maculatus* in the field pea accession PWY19.

## Materials and methods

### Plant materials

**Figure 1:**
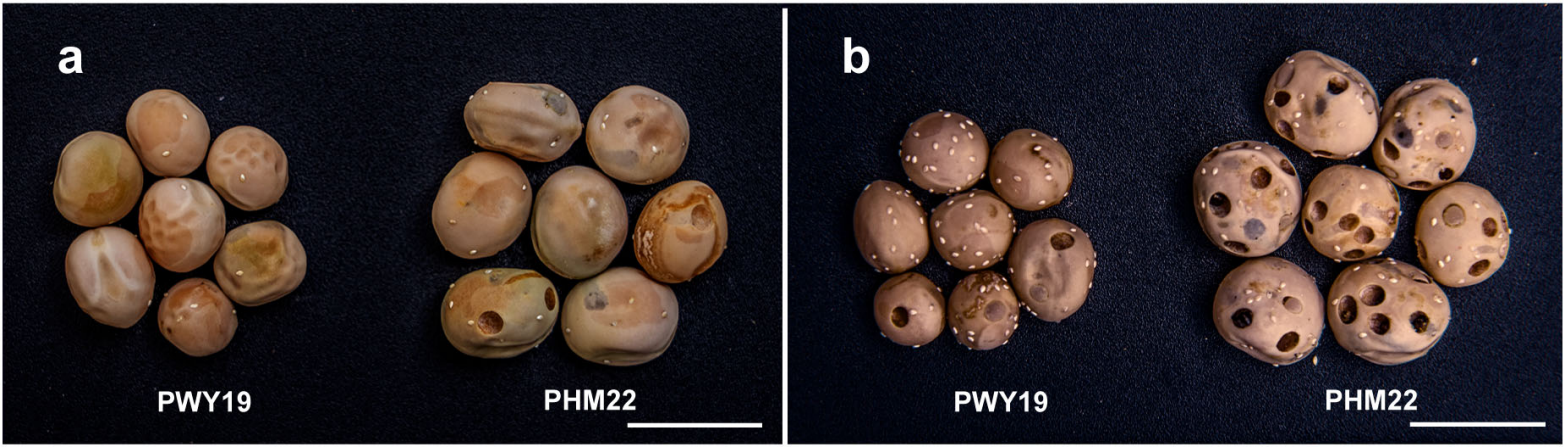
Phenotypic differences between PWY19 and PHM22 infested by *C chinensis* **(A)** and *C maculatus* **(B)**. Scale bars = 1 cm.

Three F_2_ populations (F2Y, F2N, and F2F) developed from a cross between PWY19 and PHM22 were used in this study. These F_2_ populations were derived from self-pollination of three different F_1_ plants (one population per F_1_ plant). PWY19 and PHM22 were field pea accessions provided by the Institute of Industrial Crops, Jiangsu Academy of Agricultural Sciences, Nanjing, China. Resistance/susceptibility of PWY19 and PHM22 was confirmed in this study (**Figure 1**). The population F2Y comprised 185 individuals and was planted under field conditions in April to August 2020 (spring to summer) in Youyu (latitude 39°99′N, longitude 112°47′E), Shanxi Province, China, while the population F2N consisted of 159 individuals and was planted under field conditions in November 2020 to May 2021 (winter to spring) in Nanjing (latitude 32°48′N, longitude 118°63′E), Jiangsu Province, China. The population F2F comprised 759 individuals and was grown under field conditions in April to August 2021 (spring to summer) in Youyu. The cultivation practices were the same for all populations. Briefly, soil conditions were clay loam, fertilizer applied before sowing (base fertilizer) [150 kg/ha of N:P:K (15:15:15)] and at the vegetative stage (75 kg/ha of urea) and flowering stage [150 kg/ha of N:P:K (15:15:15)], and furrow irrigation applied immediately after sowing and at the flowering and podding stages.

### Evaluation of bruchid resistance

*C. chinensis* and *C. maculatus* were used for evaluating the resistance in the populations F2Y and F2N, while only *C. chinensis* was used for evaluating the resistance in the population F2F. The bruchids were reared on susceptible mungbean seeds in boxes under constant conditions of 28°C and 70% RH at the Institute of Industrial Crops, Jiangsu Academy of Agricultural Sciences, Nanjing, China. Dry mature seeds (F_3_ seeds) produced from each F_2_ individual and the parents were used for evaluating resistance. Before the evaluation, the parental and F_3_ seeds were frozen at −20°C for more than 1 week to kill potentially contaminating bruchids from the field.

Evaluation of the resistance to these insects was conducted as per the method described by Somta et al. (2007), with slight modification. Briefly, 30 healthy seeds from each F_2_ individual were put into a small (7 × 4 cm) plastic box. Then, 30 pairs (males and females) of newly emerged bruchids (1–3 days old) were added to the box, and the insects were removed after laying eggs for 7 days. Thirty days after the introduction of insects, the numbers of seeds damaged by the bruchids (seeds with holes) were counted and converted into percentage of damaged seeds (PDS) for further analysis.

### Development of new DNA markers

To develop new DNA markers for mapping, we conducted transcriptome sequencing of pea. Total RNA was extracted from young seeds at 10, 20, and 30 days after flowering of PWY19 and PHM22 using Trizol reagent kit (Invitrogen, Carlsbad, CA, USA) and was used to prepare sequencing libraries. The transcriptome sequencing was performed using the Illumina NovaSeq6000 System (Illumina, San Diego, CA, USA) and the resulting sequences were assembled using Trinity software (Grabherr et al., 2011) by Gene Denovo Biotechnology Co. (Guangzhou, China).

The transcriptome sequences of PWY19 and PHM22 were aligned using the NCBI BLAST+2.2.31 program with an E-value cut-off of 10.0. Transcripts with insertion/deletion (Indel) of 5 bp or larger were aligned on the pea reference genome sequence (*Pisum sativum* genome assembly version 1a; Kreplak et al., 2019) to determine their locations. Subsequently, transcripts with InDels located in the genomic region containing the QTL were chosen for InDel marker development. In addition, SSRs were identified by SSR Hunter software (Li and Wan, 2005) and chosen for SSR marker development. Primers for the InDel and SSR markers were designed using the software Primer Premier 5.0 (PREMIER Biosoft, CA, USA).

### Genomic DNA extraction and DNA marker analysis

Total genomic DNA of each F_2_ individual and parents was extracted from young leaves using a modified version of the cetyltrimethylammonium bromide method (Lodhi et al., 1994). The quality and quantity of the DNA were determined using a K5800 spectrophotometer (Kaiko Technology, China).

Six hundred seventy SSR markers (Rong et al., 2020) covering all seven chromosomes of pea, together with two newly developed Indel and SSR markers (**Supplementary Table S1**), were screened for polymorphism between PWY19 and PHM22. Polymerase chain reaction (PCR) was performed in 10 μL reactions containing 25 ng of genomic DNA, 0.2 µM forward and reverse primers, and 5 µl of 2 × *Taq* Plus Master Mix II (Vazyme, Nanjing, China). The PCR was conducted in a T100 Thermal Cycler (Bio-Rad, CA, USA) programmed as follows: 95°C for 5 min; 35 cycles of 94°C for 30 s, 57°C for 30 s, and 72°C for 30 s; and finally 72°C for 10 min. The PCR products were separated in an 8% non-denaturing polyacrylamide gel (acrylamide:methylene = 19:1) and visualized by silver staining. The polymorphic markers showing polymorphism between the parents were used for genotyping the F2Y and F2N populations.

### Linkage and QTL analyses

A genetic linkage map of each population was constructed using the software QTL IciMapping 4.2 (Meng et al., 2015). A minimum logarithm of odds (LOD) value of 3.0 was used for grouping the markers. Markers were ordered using the recombination counting and ordering algorithm (RECORD) function (Van Os et al., 2005). The genetic map distance was calculated using Kosambi’s mapping function (Kosambi, 1944). The linkage map was drawn using MapChart 2.30 (Voorrips, 2002).

QTL analysis of bruchid resistance in each population was performed using the inclusive composite interval mapping (ICIM) method (Li et al., 2007) by the software QTL IciMapping 4.2. Significant LOD threshold for the QTL was determined with permutation tests with 1000 repetitions at *P* = 0.001. ICIM was performed at 0.5 cM steps.

### Narrowing down QTL region controlling bruchid resistance and identification of candidate gene for the resistance

Based on the results of QTL mapping in the populations F2Y and F2N, *qPsBr2.1* was identified as the only single major QTL for bruchid resistance (see Results). We further narrowed down this QTL region. The F_2_ individuals from the populations F2N and F2F with PDS of 0%–39% were considered to be highly resistant, while those with PDS of 81%–100% were considered to be highly susceptible. The highly resistant and susceptible F_2_ individuals were analyzed with newly developed InDel and SSR markers (**Table S1**). Subsequently, the genomic region controlling the resistance was identified by associating marker recombination and phenotype (resistant vs. susceptible). Once the genomic region for the resistance was narrowed down, genes located in the region were explored by comparison with the pea genome assembly version 1a (Kreplak et al., 2019), and a candidate gene was identified based on the function of the genes.

### Whole-genome sequencing

Whole-genome sequencing (WGS) of PWY19 and PHM22 was conducted using MGI DNBseq™ next-generation sequencing (NGS) technology (BGI, Shenzhen, China). DNA library preparation and sequencing were performed by Berry Genomics Co., Ltd. (Beijing, China), as per the manufacturer’s instructions. The sequences were filtered and *de novo* assembled into contigs using the “de_novo_assembly_illu” pipeline (Lv et al., 2016). The assembled genome sequences of the two genotypes were compared using the pea genome assembly version 1a (Kreplak et al., 2019) as a reference to find the sequence variations at candidate genes of PWY19 and PHM22.

For the re-sequencing analysis, WGS data of PWY19 and PHM22 were mapped on the pea genome sequence v.1a assembly (Kreplak et al., 2019) using the MEM algorithm of BWA (Li and Durbin, 2009). Subsequently, variant calling was performed using the “Best Practices Workflow” of GATK4 (https://gatk.broadinstitute.org). Sequence variations at candidate genes of PWY19 and PHM22 were identified from the variant call format file generated by GATK4.

### Sequence analysis of the candidate genes

Seven genes for bruchid resistance located in the *qPsBr2.1* region (see the “Results” section) were sequenced. Genomic DNA of PWY19 and PHM22 was used for PCR amplification with primers designed specifically for the genes (**Supplementary Table S1**). The PCR was conducted using KOD-FX DNA polymerase (Toyobo, Shanghai, China). PCR products were purified and sequenced.

*PsXI* (*Psat2g026280*) was selected as a candidate gene localized at *qPsBr2.1* conferring bruchid resistance (see the “Results” section). cDNA of *PsXI* was also sequenced. Rapid amplification of cDNA ends (RACE) (Frohman et al., 1988) was performed using 1 µg of RNA from roots, stems, and leaves of PWY19 and PHM22 with HiScript-TS 5′/3′ RACE Kit (Vazyme, Nanjing, China). To obtain the 5′ and 3′ ends of the *PsXI* gene, two rounds of PCR were performed using specific primers designed based on the pea genome sequence v.1a assembly (Kreplak et al., 2019) (**Table S1**). Conditions for the first and second rounds of PCR were the same as described in the manufacturer’s instructions (Vazyme, Nanjing, China). The RACE PCR products were cloned into the *pEASY* ^®^-Blunt Cloning Kit vector (TransGen, Beijing, China), and five independent clones for each end were sequenced.

All of the sequencing was performed using 3730xl DNA Analyzer (Applied BioSystems, CA, USA) by Sangon Biotech (Shanghai, China). DNA sequence alignment and analysis of protein sequence were performed using the software DNAMAN v6.0.3.99 (Lynnon BioSoft, San Ramon, CA, USA).

### Quantification of *PsXI* gene expression

PWY19 and PHM22 were grown under field conditions. Roots, leaves, and stems of young seedlings at 20 days after planting and seeds at 20 days after flowering were collected and used for the analysis of *PsXI* expression. Total RNA was extracted from roots, leaves, stems, cotyledon, seed coat, and embryo of PWY19 and PHM22 using plant RNAprep Pure kit, in accordance with the manufacturer’s protocol (Tiangen, Beijing, China). First-strand cDNA was reverse-transcribed using FastKing gDNA Dispelling RT SuperMix (Tiangen, Beijing, China).

qRT-PCR assays were performed using the ChamQ™ SYBR qPCR Master Mix reagent (Vazyme, Nanjing, China) and ABI 7500 Real-Time System (Applied BioSystem, CA, USA). The pea actin gene (NCBI accession Z25888) (Knopkiewicz and Wojtaszek, 2019) was used as an internal control for the qRT-PCR. All experiments were biologically repeated three times. Quantification of gene expression was performed by the 2^−ΔΔCT^ method (Livak and Schmittgen, 2001). Primers used for the qRT-PCR analysis are listed in **Table S1**.

### Subcellular localization of PsXI^-PWY19^ and PsXI^- PHM22^

The coding sequences (CDSs) of *PsXI^-PWY19^* and *PsXI^-PHM22^* (see the “Results” section) without the stop codon were cloned into the binary vector pCAMBIA1305.1-GFP, under control of the CaMV35S promoter. The primers PsXI-F1/PsXI-R and PsXI-F2/PsXI-R (**Supplementary Table S1**) with *XbaI* and *BamHI* restriction sites were used for subcloning the CDSs of *PsXI^-PWY19^* and *PsXI^-PHM22^* without the stop codon, respectively. Two fusion constructs, PsXI^-PWY19^-GFP and PsXI^-PHM22^-GFP, as well as pCAMBIA1305.1-GFP as a control, were infiltrated into the abaxial side of 4-week-old *Nicotiana benthamiana* leaves. After 48–72 h of incubation, fluorescence signals were observed using a Zeiss LSM 880 confocal microscope (Leica Microsystems, Germany). AtPP2A-mCherry and AHL22-mCherry were used as plasma membrane and nuclear markers, respectively.

## Results

### Variation of *C. chinensis* and *C. maculatus* resistance in F_2_ populations

PWY19 and PHM22 contrasted in their responses to *C. chinensis* and *C. maculatus* (**Figure 1**). The percentages of damaged seeds (PDSs) caused by *C. chinensis* and *C. maculatus* in PWY19 were 6.97% and 30%, whereas those in PHM22 were 93.33% and 100%, respectively. F_2_ populations F2Y and F2N of the cross PWY19 × PHM22 were evaluated for resistance to *C. chinensis* and *C. maculatus*. In all cases, the PDSs varied between 0% and 100%. The mean PDSs caused by *C. chinensis* and *C. maculatus* in the population F2Y were 33.57% and 37.73%, while those in the population F2N were 41.32% and 65.61%, respectively. In both populations, the correlation between PDSs caused by *C. chinensis* and *C. maculatus* was high and significant, being 0.83 (*P* < 0.0001) for F2Y and 0.70 (*P* < 0.0001) for F2N.

**Figure 2:**
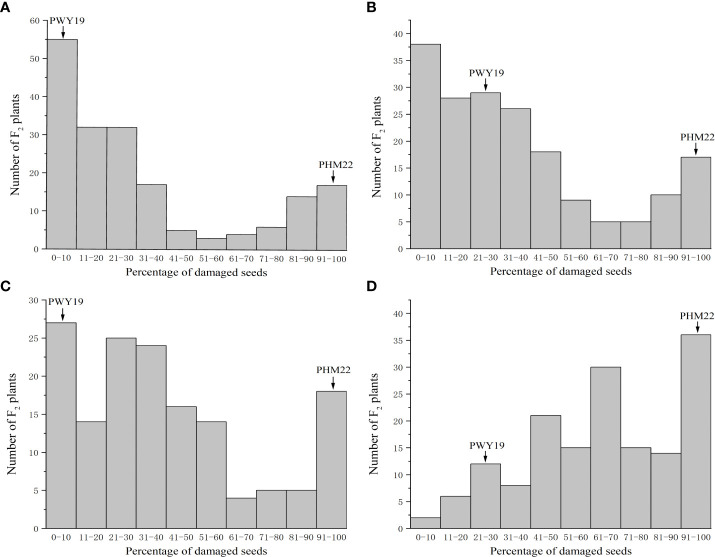
Frequency distribution of percentage of damaged seeds caused by *C chinensis* **(A)** and *C maculatus* **(B)** in the F_2_ population F2Y comprising 185 individuals grown in Youyu, and *C chinensis* **(C)** and *C maculatus* **(D)** in the F_2_ population F2N consisting of 159 individuals grown in Nanjing. The F_2_ populations are derived from a cross between PWY19 and PHM22.

The frequency distribution of the PDSs caused by *C. chinensis* and *C. maculatus* in the populations F2Y and F2N is shown in **Figure 2**. The distribution of PDSs caused by *C. chinensis* in both populations was bimodal (**Figures 2A, C**), as was the distribution of PDSs caused by *C. maculatus* in F2Y (**Figure 2B**), but that in F2N was not bimodal (**Figure 2D**).

### QTL analysis of bruchid resistance

Among 670 SSR markers screened for polymorphism between PWY19 and PHM22, 126 markers (18.81%) showed unambiguous polymorphism (**Table S1**). These polymorphic markers together with a genic marker (see section “Sequence Variations of the Candidate Gene *PsXI*”) were used to analyze the F_2_ populations F2Y and F2N (**Figure S1**). A genetic map constructed for the population F2Y comprised eight linkage groups with a total length of 1,313.1 cM and an average interval distance between adjacent markers of 10.5 cM (**Figure S2**). The genetic map constructed for the population F2N consisted of eight linkage groups with a total length of 1,148.3 cM and an average interval distance between adjacent markers of 9.2 cM (**Figure S3**). In general, the linkages and orders of the markers in the two maps were consistent.

**Figure 3:**
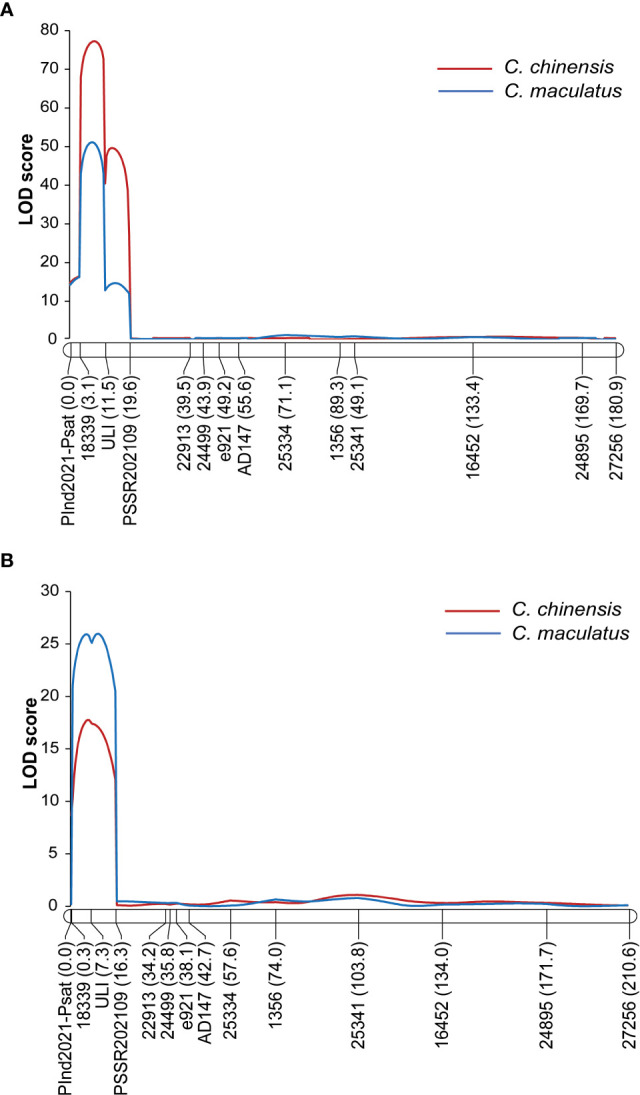
LOD graph of the QTL *qPsBr2.1* in LG2 detected for the percentage of damaged seeds caused by *C chinensis* and *C maculatus* in the F_2_ population F2Y grown in Youyu **(A)** and in the F_2_ population F2N grown in Nanjing **(B)**. The F_2_ populations are derived from a cross between PWY19 and PHM22.

QTL analysis for the resistance to *C. chinensis* and *C. maculatus* in F2Y identified two closely linked QTLs for the *C. chinensis* resistance and one major QTL for the *C. maculatus* resistance, while that in F2N identified a single major QTL for the resistance to both bruchids (**Table 1**). In all cases, the QTLs were located on LG2 and the genic marker ULI was the most closely linked to the QTLs (**Table 1** and **Figure 3**). Depending on the population and bruchid species, phenotypic variance explained (PVE) by the QTL was between 26.42% and 62.60%, the additive effect varied between −17.27% and −40.86%, and the dominant effect varied from −2.52 to −21.12. The alleles from PWY19 decreased the PDS. Since the QTLs were consistently identified at the same interval with similar genetic effects for different bruchid species across different populations grown in different environments, we considered that these QTLs were the same locus and designated this locus *qPsBr.*

**Table 1:** Summary of the QTLs associated with bruchid resistance across different locations identified by inclusive composite interval mapping in the pea F_2_ populations of the cross PWY19 × PHM22.

Population	Bruchid species	QTL name	Linkage group	Position (cM)	Marker interval	LOD	PVE (%)	Additive effect	Dominant effect
F2Y	*C. chinensis*	*qPsBr2.1*	2	8.0	18339 - ULI	77.22	52.11	-40.86	-20.73
		*qPsBr2.2*	2	14.0	ULI - PSSR202109	19.63	26.42	-27.20	-21.12
	*C. maculatus*	*qPsBr2.1*	2	7.0	18339 - ULI	51.11	61.35	-36.82	-15.85
F2N	*C. chinensis*	*qPsBr2.1*	2	6.0	18339 - ULI	17.76	62.60	-21.26	-13.91
	*C. maculatus*	*qPsBr2.1*	2	9.5	ULI - PSSR202109	25.97	53.30	-27.71	-2.52

LOD, logarithm of the odds; PVE, percentage of variance explained by the QTL.

### Narrowing down the *qPsBr* region

**Figure 4:**
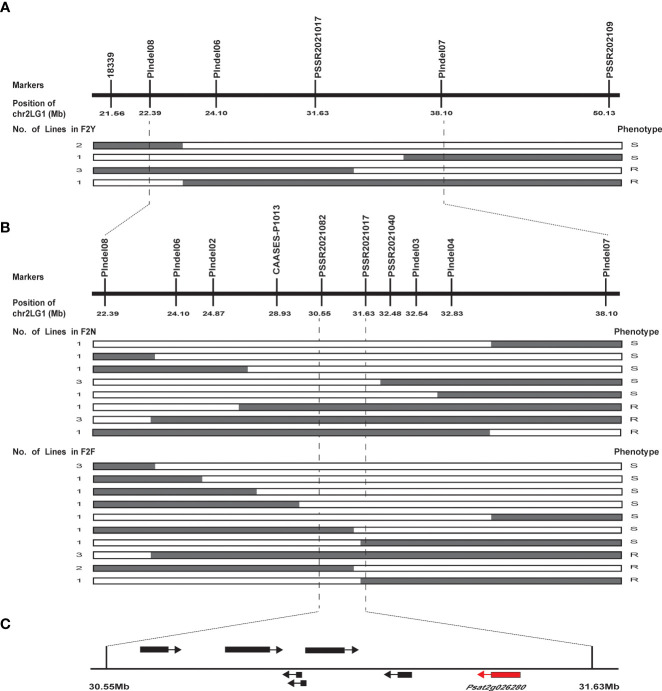
Fine mapping of *qPsBr2.1* controlling seed resistance to *C chinensis* and *C maculatus* in field pea cultivar PWY19. **(A)** *qPsBr2.1* is mapped onto pea chromosome 2 between the InDel markers PIndel08 and PIndel07 using the F_2_ population F2Y. The numbers under the markers represent the positions of the markers on chromosome 2. **(B)** *qPsBr2.1* is narrowed down to marker interval PSSR2021082–PSSR2021017 using the F_2_ populations F2N and F2F. White and gray boxes in the recombinants indicate homozygous genotype of the susceptible parent and heterozygous genotype, respectively. **(C)** A 1.07 Mb genomic region harboring *qPsBr2.1*. Based on the pea reference genome sequence database (*Pisum sativum* v1a), this region contains seven annotated genes.

We narrowed down the genomic region of *qPsBr* by selectively genotyping F_2_ plants in the F_2_ populations F2N and F2F. Given that the rate of damage of F_2:3_ seeds caused by *C. chinensis* in two different environments showed a bimodal distribution, C. *chinensis* resistance was selected as the target trait for fine mapping of the *qPsBr* locus. According to the frequency distribution of the rate of seed damage caused by *C. chinensis* in F2Y and F2N (**Figures 2A, C**), the PDSs from 40% to 80% were removed, while the remaining plants with an extreme phenotype were used for fine mapping. Four polymorphic markers (PIndel08, PIndel06, PSSR2021017, and PIndel07) from the candidate region were selected to map *qPsBr* in F2Y and seven recombinant individuals between PIndel08 and PIndel07 were screened (**Figure 4A**). The results revealed that *qPsBr* is located between PIndel08 and Pindel07. The markers Pindel08 and Pindel07 together with additional markers were used to genotype the populations F2N and F2F for the fine mapping of *qPsBr*. By associating the marker genotypes with the bruchid resistance phenotypes, the *qPsBr* locus was narrowed down to the genomic region between markers PSSR2021082 and PSSR2021017 (**Figure 4B**).

Based on the reference pea genome sequence, the markers PSSR2021082 and PSSR2021017 were 1.07 Mb apart on chromosome 2 (chr2LG1). They were at positions 30559247 bp and 31635279 bp, respectively. There are seven annotated genes in this region (**Figure 4C**; **Table 2**). Sequences of these genes were analyzed using Sanger sequencing or NGS whole-genome resequencing. Sequence variations between PWY19 and PHM22 affecting protein coding were found in all of these annotated genes, except for *Psat2g026120* (**Table S2**). Therefore, the gene related to bruchid resistance could not be confirmed from the sequence variations. Nonetheless, based on the functions of the predicted genes (**Table 2**), *Psat2g026280* encoding a xylanase inhibitor N-terminal was considered as a candidate gene for bruchid resistance. *Psat2g026280* was selected because previous studies showed that enzyme inhibitors are involved in resistance to *C. chinensis* and *C. maculatus* (Ishimoto and Kitamura, 1989; Zhang et al., 2021). The gene *Psat2g026280* was designated *PsXI.*

**Table 2:** Annotated genes in mapping region of *qPsBr2.1*.

Gene ID	Chromosome	Location	Description
*Psat2g026040*	2	30986483.30988524 (+ strand)	Ankyrin repeats (3 copies)
*Psat2g026080*	2	31151770.31154995 (+ strand)	Domain of unknown function
*Psat2g026120*	2	31183831.31184236 (- strand)	Bacterial Fmu (Sun)/eukaryotic nucleolar NOL1/Nop2p
*Psat2g026160*	2	31189339.31189734 (- strand)	Unknown gene
*Psat2g026200*	2	31189341.31192165 (+ strand)	Unknown gene
*Psat2g026240*	2	31342741.31343754 (- strand)	Ubiquitin-conjugating enzyme/RWD-like
*Psat2g026280*	2	31546727.31548839 (- strand)	Xylanase inhibitor N-terminal

### Sequence variations of the candidate gene *PsXI*

Whole-genome sequencing of PWY19 and PHM22 was performed by NGS. For PWY19, 1.55 billon reads with 231.92 Gb were generated and 1.54 billion reads with 230.85 Gb were filtered and *de novo* assembled into 6,287,918 contigs. In the case of PHM22, 1.68 billon reads with 251.54 Gb were produced and 1.67 billion reads with 250.28 Gb were filtered and *de novo* constructed into 6,403,370 contigs. Sequence homology search revealed that the *Psat2g026280* sequence matched to three contigs: 163824256, 163565986, and 163842058 of PWY19 (**Figure S4**). Sequence alignment showed that partial insertion sequences of these contigs did not exist in the reference sequence and there were InDel polymorphisms in the intron of *Psat2g026280.*

**Figure 5:**
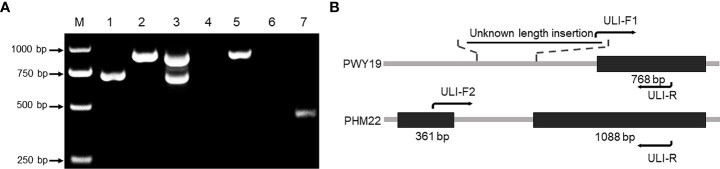
Variation of *PsXI* gene between PWY19 (bruchid-resistant) and PHM22 (bruchid-susceptible). **(A)** PWY19 (lane 1), PHM22 (lane 2), and their F_1_ hybrid (lane 3) were amplified using sequence-specific primers ULI-F1/ULI-F2/ULI-R. Lanes 4 and 5 represent amplification from genomic DNA of PWY19 and PHM22 using ULI-F2/ULI-R, respectively. Lanes 6 and 7 represent amplification from cDNA of PWY19 and PHM22 using ULI-F2/ULI-R, respectively. **(B)** Structure of *PsXI* in PWY19 and PHM22. Black boxes represent coding regions.

Three primers, ULI-F1/ULI-F2/ULI-R, were designed to amplify specific alleles of *PsXI*. One of them, ULI-F1, was aligned on the partial insertion sequence that did not exist in the reference sequence (**Figure 5B**). Genomic DNA of PWY19, PHM22, and their F_1_ hybrid was amplified using the combination of the three primers. The results showed that allele-specific bands of 709 bp and 883 bp were generated from PWY19 and PHM22, respectively. Meanwhile, co-dominant bands were amplified from the F_1_ hybrid (**Figure 5A**). Nonetheless, when the genomic DNA and cDNA of PWY19 and PHM22 were amplified with the primer pair ULI-F2/ULI-R, PCR generated the expected product from PHM22 but failed to generate a product from PWY19 (**Figure 5A**). Therefore, we supposed that there is an insertion of unknown length in the intronic region of PWY19 that results in sequence difference between PWY19 and PHM22 and a change in transcript of PWY19 (**Figure 5B**). The polymorphism of the marker “ULI” was verified in the F2Y and F2N populations and used for gene mapping (**Figures 3**, **S1**, **S2**).

**Figure 6:**
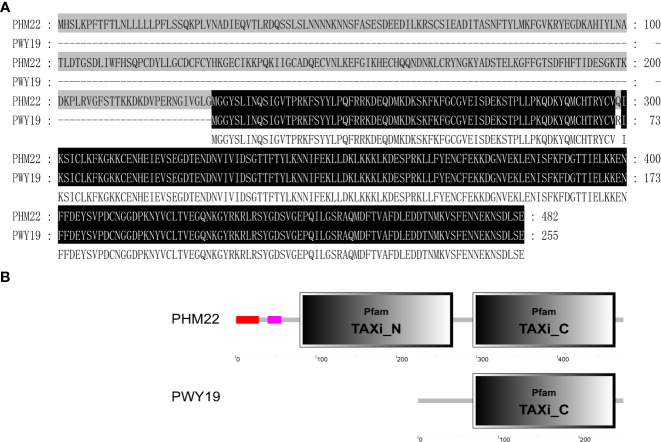
Variation of deduced protein of PsXI. **(A)** Alignment of the deduced protein sequences encoded by *PsXI* in PWY19 and PHM22. **(B)** Domain architecture analysis of PsXI in PWY19 and PHM22.

We obtained the full-length cDNA sequence of the *PsXI* gene in PWY19 and PHM22 by RACE-PCR. Sequence alignment of the deduced PsXI sequences from PWY19 and PHM22 is shown in **Figure 6A**. Based on BLAST and SMART (https://smart.embl.de; Letunic et al., 2021) analyses, the open reading frame of *PsXI* in PHM22 encodes a protein of 482 amino acids, and the deduced *PsXI* protein comprises a predicted signal peptide, predicted TAXI (*Triticum aestivum* xylanase inhibitor) N-terminal, and predicted TAXI C-terminal structure (Fierens et al., 2003). The open reading frame of *PsXI* in PWY19 lacks the first 227 amino acids compared with PHM22, and encodes a TAXI C-terminal domain (**Figure 6B**).

### Expression analysis and subcellular localization of *PsXI* gene

**Figure 7:**
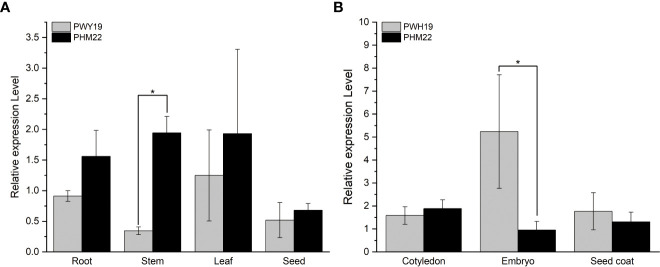
Relative expression levels of *PsXI* in PWY19 and PHM22. **(A)** Expression levels of *PsXI* in different tissues. **(B)** Expression levels of *PsXI* in cotyledon, embryo, and seed coat of seeds at 20 days after flowering. Three biological replicates were used to produce average expression levels. The asterisks (*) represent significant differences (*P* < 0.05) as determined by Student’s t-test.

Relative expression levels of *PsXI* in different tissues in PWY19 and PHM22 were quantified using qRT-PCR. The gene exhibited low expression in the root, stem, leaf, and seed. The transcript level of *PsXI* in stem was significantly higher in PHM22 than in PWY19 (**Figure 7A**). In the different tissues of seed at 20 days after flowering, the levels of *PsXI* transcripts in embryo were significantly higher in PWY19 than in PHM22 (**Figure 7B**).

**Figure 8:**
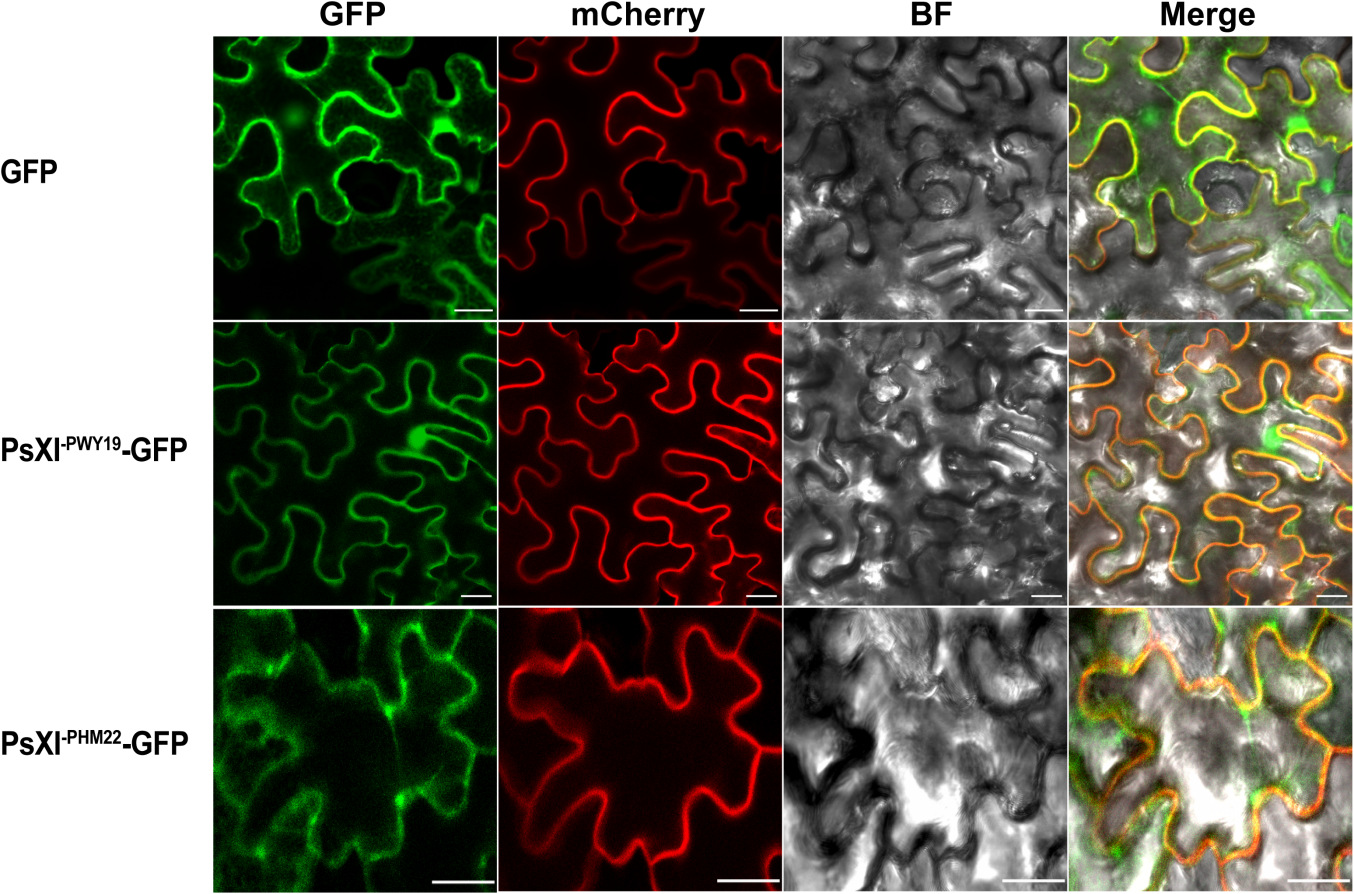
Subcellular localization of PsXI-GFP fusion proteins. GFP was used as a control. mCherry channel acts as a marker of the plasma membrane. BF, bright field. Scale bars, 20 μm.

We examined the subcellular localization of the PsXI^-PWY19^-GFP and PsXI^- PHM22^-GFP fusion proteins in *Nicotiana benthamiana* leaves. The PsXI^-PWY19^-GFP fusion was co-localized with plasma membrane marker and nuclear marker and showed no difference from the control plasmid that only encoded GFP protein, whereas the PsXI^- PHM22^-GFP fusions were mainly localized in the plasma membrane in *Nicotiana benthamiana* foliar cells (**Figures 8**, **S5**). Evaluation of the subcellular localization showed that the *PsXI* of PWY19 and PHM22 was targeted to different locations.

## Discussion

*C. chinensis* and *C. maculatus* are destructive stored product insects that infest field pea (Bhagwat et al., 1995; Umrao and Verma, 2002; Duan et al., 2014; Teshome et al., 2015; Sharma et al., 2016; Aznar-Fernández et al., 2017). In this study, we reported for the first time on the genetics of the resistance of field pea to *C. chinensis* and *C. maculatus*. Our QTL mapping clearly demonstrated that the seed resistance to these bruchid species in field pea cultivar PWY19 is controlled by a single major locus (**Table 1** and **Figures 2**, **3**). This is supported by the high and significant correlation between PDSs caused by the two bruchid species. The high additive effect and PVE of the QTL *qPsBr2.1* together with its stable expression across environments (**Table 1**) suggest that this QTL will be useful for breeding bruchid-resistant pea cultivars.

Although the resistance of PWY19 to *C. chinensis* and *C. maculatus* is controlled by a single major QTL with a large genetic effect and thus breeding for resistance would not be difficult, testing for bruchid resistance is time-consuming and resource-intensive, and must be performed under legal and ethical research standards involving insects. The use of marker-assisted selection (MAS) can overcome these limitations and difficulties. An example of MAS of bruchid resistance was reported and reduced the time and resource demands for the development of bruchid-resistant cultivars in mungbean [*Vigna radiata* (L.) Wilczek] (Wu et al., 2022). In our study, the primers/marker ULI-F1/ULI-F2/ULI-R could detect *PsXI* polymorphism and would be ideal for MAS of *PsXI* for the resistance of field pea to *C. chinensis* and *C. maculatus*.

Previously, QTLs for resistance to the bruchid species *B. pisorum* were identified: three major QTLs on LGs 2, 4, and 5 for cotyledon resistance and two major QTLs for pod wall/seed coat resistance on LG2 and LG5 in wild pea *P. fulvum* accession ATC113 (Aryamanesh et al., 2014), and four QTLs for seed resistance on LGs I, II, and IV in wild pea *P. sativum* ssp. *syriacum* accession P665 (Aznar-Fernández et al., 2020). BLASTN search of the markers associated with these QTLs revealed that the location of *qPsBr2.1* differs from the QTLs for *B. pisorum* resistance. This suggests that different resistance mechanisms exist for different bruchid species in pea germplasm. Pyramiding these QTLs into a pea cultivar would provide broad-spectrum resistance to bruchid species.

The *qPsBr2.1* locus was narrowed down to a 1.07-Mb region on chromosome 2 of the pea reference genome (**Figure 4B**). Based on the pea reference genome, this 1.07-Mb region harbored only seven annotated genes. The genome of pea has low gene density, of approximately 11 genes/Mb (44,756 genes in a 3.92 Gb genome assembly) (Kreplak et al., 2019). Thus, it is rational that only seven genes were annotated in our mapping region of *qPsBr2.1*. Among these seven genes, *PsXI* (*Psat2g026280*) was predicted to encode a xylanase inhibitor and was selected as a candidate gene for the resistance at *qPsBr2.1*. Proteinase inhibitors are a major class of biochemicals that plants employ to defend themselves against herbivorous insects (Lawrence and Koundal, 2002; Huma and Khalid, 2007) and fungi (Brito et al., 2006; Tundo et al., 2020). Bruchids are phytophagous insects that consume starchy seeds. They use various enzymes including glycoside hydrolases (GHs) to digest seed starch/carbohydrate to obtain energy and nutrients for growth and development. α-Amylase inhibitor isolated from common bean (*Phaseolus vulgaris* L.) has been shown to completely inhibit the growth and development of *C. chinensis*, *C. maculatus*, and *Zabtotes subfasciatus* Boheman (Ishimoto and Kitamura, 1989). The α-amylase inhibitor I gene from common bean has been used to produce transgenic pea resistance to *B. pisorum* (Morton and Schroeder, 2000). Mungbean genes *VrPGIP1* and *VrPGIP2* each encoding a polygalacturonase inhibitor were also reported to confer resistance to *C. chinensis* and *C. maculatus* (Chotechung et al., 2016; Kaewwongwal et al., 2017; Kaewwongwal et al., 2020; Zhang et al., 2021). Endo-β-1,4-xylanase, an enzyme belonging to the GH5_10 subfamily and having enzymatic activity of degrading xylan, has been identified in *C. caculatus* (Busch et al., 2017). This enzyme is expressed in the gut and has digestive function in *C. maculatus* (Busch et al., 2017). Xylan is a polysaccharide made from xylose residues. Therefore, xylanase inhibitors (e.g., *PsXI*) can limit food digestion by xylanases in *C. maculatus* and possibly other bruchid species, and confer bruchid resistance.

*PsXI* sequence analysis in the mapped parents revealed polymorphisms causing changes of protein coding in *PsXI* of PWY19. A long insertion (albeit with an unknown specific length) in the intron of *PsXI* prevents the two exons of the gene from joining as one ORF in PWY19. However, we failed to amplify this insertion irrespective of whether direct PCR or genomic walking was used. The results of qPCR showed that there was no significant difference between PWY19 and PHM22 in the expression levels of the original second exon of *PsXI* in several tissues, indicating the presence of a promoter or cis-acting element in this insertion of unknown length. The changes of protein coding in *PsXI* of PWY19 provided a hint of the functional difference associated with bruchid resistance.

No significant difference was detected in the expression levels of *PsXI* in seeds and cotyledons (the major parts of seeds) between PWY19 and PHM22, indicating that the expression level in the tissue ingested by bruchids is not responsible for the resistance. The higher expression level of *PsXI* in the embryos of bruchid-resistant PWY19 suggested that embryos would be the arsenal of PsXI. Besides, the different patterns of subcellular localization between *PsXI^-PWY19^* and *PsXI^-PHM22^* may result in different protein translocation in tissues. We hypothesized that a process occurs in which *PsXI* is expressed in the embryos of PWY19 and the protein is translocated to other parts of the seeds.

The results of fine mapping indicated that *PsXI* is an important candidate gene for the resistance to *C. chinensis*. Besides, QTL analysis revealed that a major locus in the marker interval of 18339 and PSSR202109, which contains *PsXI*, is responsible for the resistance to both *C. chinensis* and *C. maculatus*. Thus, we assumed that *PsXI* is a pleiotropic gene that controls resistance to both *C. chinensis* and *C. maculatus*. However, more evidence is needed to verify the relationship between *PsXI* and bruchid resistance in PWY19. Nonetheless, our findings are the first line of evidence showing an association between insect resistance and plant xylanase inhibitor.

## Data availability statement

The transcriptome sequences generated in this study are deposited to the National Genomics Data Center (https://ngdc.cncb.ac.cn) (Project accession number "PRJCA012315").

## Author contributions

PS, XC, and XZ conceived idea and designed the study. JC, YL, XY, and ZZ supervised the study. JY, JC, YL, XY, and ZZ performed phenotyping, genotyping, DNA sequencing, expression analysis, subcellular localization analysis, and bioinformatics analysis. JY and PS conducted data analysis. JY and JC wrote manuscript. XZ, YZ, CX, and PS revised manuscript and secured research funds. All authors contributed to the article and approved the submitted version.
