# Supplementary material for: Mapping of quantitative trait locus reveals PsXI gene encoding xylanase inhibitor as the candidate gene for bruchid (Callosobruchus spp.) resistance in pea (Pisum sativum L.)
Source: Front Plant Sci. 2023 Jan 30;14:1057577. doi: 10.3389/fpls.2023.1057577 (PMC9923024; doi:10.3389/fpls.2023.1057577)

Figure S1. Agarose gel electrophoresis showing DNA band patterns detected by the genic marker "ULI" in some individuals of pea F_2 populations F2Y and F2N derived from the cross between PWY19 and PHM22.

a

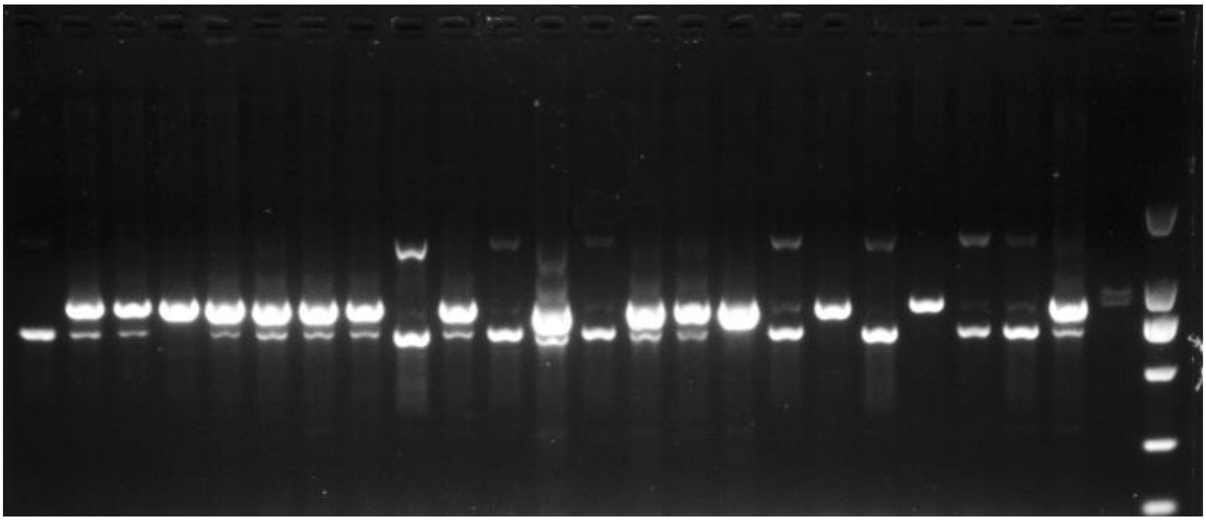

b

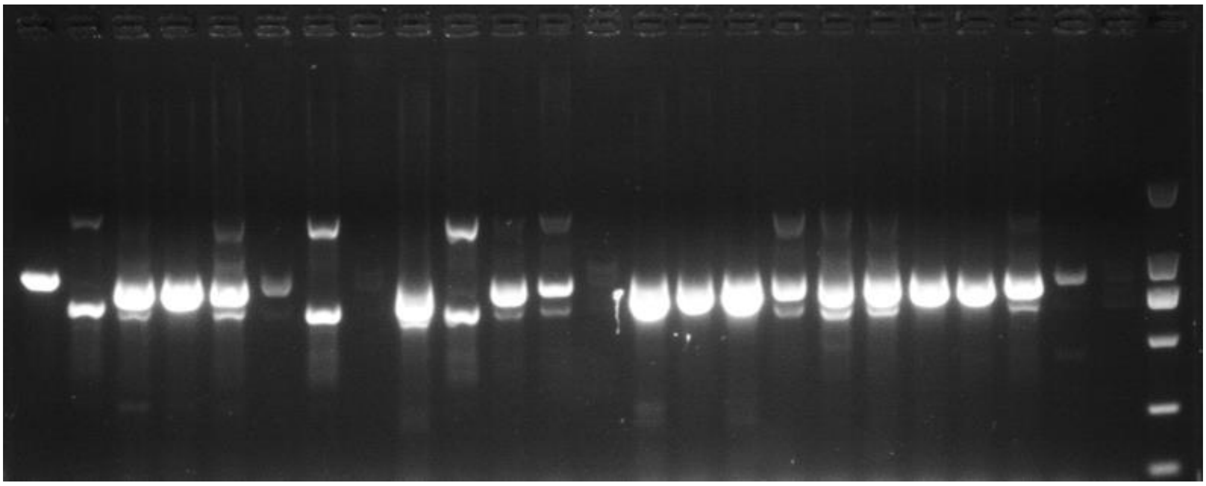

Supplement: Supplementary file 1 [file DataSheet_1.pdf]
