# Supplementary material for: Mapping of quantitative trait locus reveals PsXI gene encoding xylanase inhibitor as the candidate gene for bruchid (Callosobruchus spp.) resistance in pea (Pisum sativum L.)
Source: Front Plant Sci. 2023 Jan 30;14:1057577. doi: 10.3389/fpls.2023.1057577 (PMC9923024; doi:10.3389/fpls.2023.1057577)

Supplementary Figure S2. Genetic linkage map of field pea F_2 population of 185 individuals derived from a cross Woyaowandou × Haimenbiahua. The map is constructed with 126 simple sequence repeat markers. The population was grown in Youyou, Shanxi, China

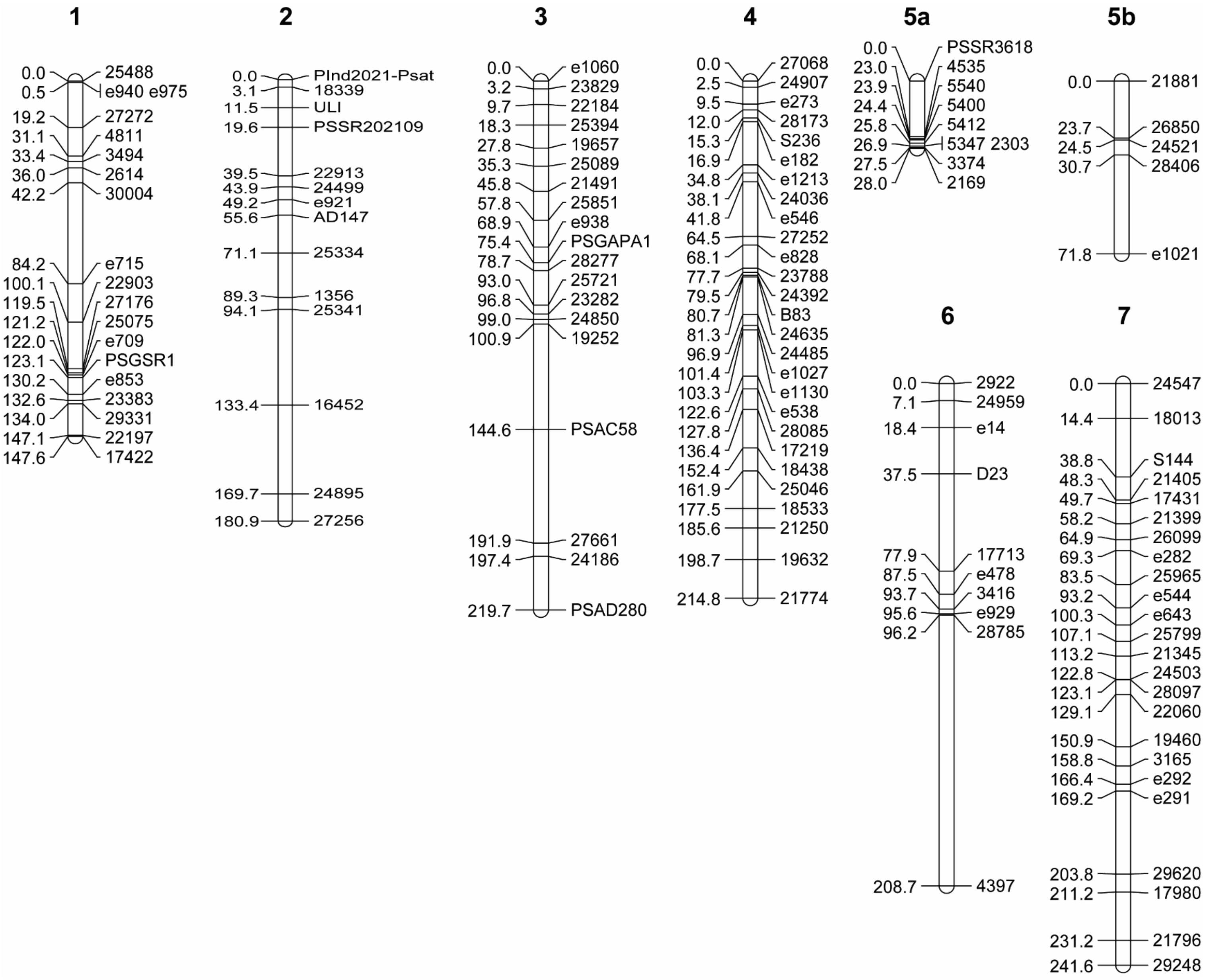

Supplement: Supplementary file 2 [file DataSheet_2.pdf]
