# Supplementary material for: Mapping of quantitative trait locus reveals PsXI gene encoding xylanase inhibitor as the candidate gene for bruchid (Callosobruchus spp.) resistance in pea (Pisum sativum L.)
Source: Front Plant Sci. 2023 Jan 30;14:1057577. doi: 10.3389/fpls.2023.1057577 (PMC9923024; doi:10.3389/fpls.2023.1057577)
Supplement: Supplementary file 4 [file DataSheet_4.pdf]

Supplementary Figure S4. Contigs of PWY19 matched to *Psat2g026280*. Letters with yellow background represent those sequences matched to ORF of *Psat2g026280*. Letters with red background represent those sequences did not exist in the reference genome of pea

>163565986
TTTTTTTTCCTTCCTCTCTAGTAAATTGCTCTAAATTTCTCTCCTCATAACATGCAGGTCTAATCACATCAAAAATCATAAAAGCAATACCTTAGAAGCCCTACTT
AACGTGGGGATTAATTCTCTATCCTTTCCGAATGTTTAACACTTTAGAATTTATGCAAATATGCTAATCCAAATTATCCTCACTCAATGAATAGACACAAGCCATT
TCACTTATCTCTATGTTGCTAGCTTCTCTATAAATAACAACTTATCCATCATTAAATTTCCATGCATGCATTAGTTCCACTACTAATACTACTACTACTACTCTTGCA
AATCCTAAAATGCATTCCCTAAAACCATTCACTTTTACACTAAATCTATTACTCTTGCTACCATTTCTTTCATCCCAAAAACCCTTGGTCGATGCTGACATCGAG
CAAGTTACTCTCCGTGACCAATCATCACTCTCTCTGAACAACAACAACAAAAACAACTCTTTTGCATCTGAATCTGATGAAGAAGACATTTTGAAAAGAAGTT
GTTCCATTGAAGCTGATATAACTGCATCTAGTTTCACCTACCTTATGAAATTCGGTGTTAAAAGGTACGAAGGCGATAAAGCTCACATATATTTAAATGCCACTC
TAGACACCGGAAGCGATCTCATATGGTTTCATAGCCAGCCTTGCGATTATCTACTAGGTAAATTCAGTTTGTTTTTATTTTTAGATAAATTATTTGACAATTTGATC
TTTTTTTCAGTGATAAATATTCTATCTCCGAAAAAAAAAA

>163842058
AAAAAAAAAAGAATAGGAAAATGTTAACATGATTCTTAATTACTTGTAGAAATATTCTGTTTAAATGTAGGTATTTAATTTTTCAAAATTTCAAAAAATACTCTA
TTAACTTTTATATTTTTAGTTTTACCATTTTTAACATATGCTTTGAAAACACAAGTTAGCACGATTTTAGTGAATTAAAATTTGTTAAAATAGTGAGGTCCACTATT
AAATCGGTCCATAGTAAACGTTCCTTGTAAAACAACTTTTATTTTGATATAGTTATTTTCATAGTGAAATATCACATGTTATTTTAAATTTTTGTTATTATAAAATTC
ACTTTTAGAAAAAGTAGTAAGAGTTCATGTTTGTACAATTTTTTAAAAGGTGATGTCAAATTGATAGTGGGAGTTTTTGACTAAGAAATATTGATTATTAAGAG
ACTTTTTTATTGAGGGAGTTTTTGACTAGGAAACATTGATTATTGTGAGACTTCTTCATTTAGAGTAATACCTTTATGAGAATACCATATATTCATCAAGAATCTT
AAGGTAATAAGTCAGTGAGCTCTATCATTTATAAATGCTCAAGTCTCCCTATTTTCAACTAATGTGAGATTATTATCCACACTACTCACTCTACAAAAATTGATTC
ATTTTTACAATACTAAAGAAATAATAATTATAGTTTTTCTTTATTATACTATTAAGTATTTTCTTTAATGTTCTTTTTATTATTATTATTATTTTTATAGTATTAATGAA
GACTTTTTTATATAAAAAATTAATAATTTTTTTTTCATACAAAATTAATTATAACTTTTGATATTTGTAAAGTTTATAAAGTGAGTCGGAAAGATTCAACATAATTA
ATAATGAGATATATGATTGTAACAGCGTGGAATCAAATTCTGGGATTCAATCTATTCATTTTTAAAGAAAATATAATACAATGCGAATTGAGTTTTGCTGATGACT
ACATAAATTACAAAAAAGAATAATTATAGAAAAAGATTTTAATATAATCCATTTATTTTATTGAATCAAAATTTAATGGTCAAGATTAATTATTCTAATTTTTCAAA
ATAACCAAGTCTTTTCACTTGAGTAGCTAATAATACAAAAAAGGCTCTAAAGGTAGCTTGTAAATGCATCGTAGGTACTAATGCAACTCAACAAATAACAGAA
AGAAAAAAAAGACTATACAATACAACATACCAAAATAAATTAATCAATGGTTATTAAAATTAATTTAGTAAATATTTTATTCTCAAAATTTATCAATAATTAATCAT
TAACAATATTTATTTGAAGAATGATTTACTAGTTATTCTTGTTTTTCACTATAACCCATGTTATATCTTTTCTTATGAATTTTTTTTTAAAATATCCAAAAATTTAAA
AAAAATTTCAAA

>163824256_RevCom
TTGAAATTTTTTTTAAATTTTTGGATATTTTAAAAAAAAATTCTTTTCTTATCACCAACCCTTAAGAAAATCTCTGCATGGAAATCATCTTTATTCATATTCTTA
GAAATAATAATAATTTATTTATTTTTAATTAGAATCCTTGAGTGGTGGTTATAACGAAAGAAAAGAGAGTAAATTTTATTTTTATTCTTTAATTAACAAAAGAA
ATTATGATTTATTGTTTGTAAAAACTACATGTGTTTGTTTCTATCTAAATTTTTTTCCTAACCCTTATATCTTTTGTCTTGTTATTGTATTACGTTACAGGTTGT
GATTGTTTTTGTTATCACAAAGGTGAATGTATCAAGAAGCCCCAGAAAATCATCGGTTGTGCAGATCAAGAATGTGTGAACTTGAAAGAATTTGGAATCAA
ACATGAATGTCACCAGCAAAATGATAACAAATTATGCCGGTACAACGGTAAATATGCAGACAGTACTGAATTAAAAGGATTTTTTGGTACTTCAGATTTTCA
TTTTACAATTGACGAAAGTGGTAAAACCAAAGATAAACCATTGAGAGTTGGATTTTCGACCACAAAGAAGGATAAAGATGTACCAGAAAGAAATGGAATTG
TTGGACTTGGAATGGGAGGATACTCTTTGATAAATCAATCTATAGGTGTAACCCCGAGGAAATTCTCTTATTATCTTCCTCAATTTCGTCGGAAAGATGAA
CAAGATATGAAAGATAAAAGCAAGTTCAAATTTGGTTGCGGTGTGGAAATTTCGGATGAAAAAAGTACTCCTTTGTTACCAAAACAAGACAAATATCAAAT
GTGCCACACTCGTTACTGCGTCCGAATTAAAAGTATCTGTCTGAAATTTAAAGGCAAAAAATGTGAAAATCATGAGATAGAAGTGAGTGAAGGTGACACT
GAGAATGATAATGTAATAGTTATTGATTCAGGTACTACATTTACGTATCTTAAAAACAATATATTCGAAAAATTATTAGATAAACTAAAAAAGAAGTTAAAGG
ACGAAAGCCCAAGAAAGTTATTATTTTATGAAAATTGTTTTGAGAAGAAGGATGGGAATGTTGAGAAATTGGAGAATATATCATTTAAATTTGATGGTACAA
CAATTGAATTGAAGAAGGAGAATTTCTTTGATGAATATAGTGTTCCAGATTGCAATGGTGGAGACCCAAAAAACTATGTTTGCTTGACAGTGGAGGGACA
AAATAAAGGGTATCGTAAAAGGTTGCGTAGCTATGGAGACTCAGTGGGAGAACCACAAATACTTGGAAGTAGGGCACAAATGGATTTTACGGTTGCTTTT
GATCTTG
