# Supplementary material for: Mapping of quantitative trait locus reveals PsXI gene encoding xylanase inhibitor as the candidate gene for bruchid (Callosobruchus spp.) resistance in pea (Pisum sativum L.)
Source: Front Plant Sci. 2023 Jan 30;14:1057577. doi: 10.3389/fpls.2023.1057577 (PMC9923024; doi:10.3389/fpls.2023.1057577)
Supplement: Supplementary file 5 [file DataSheet_5.pdf]

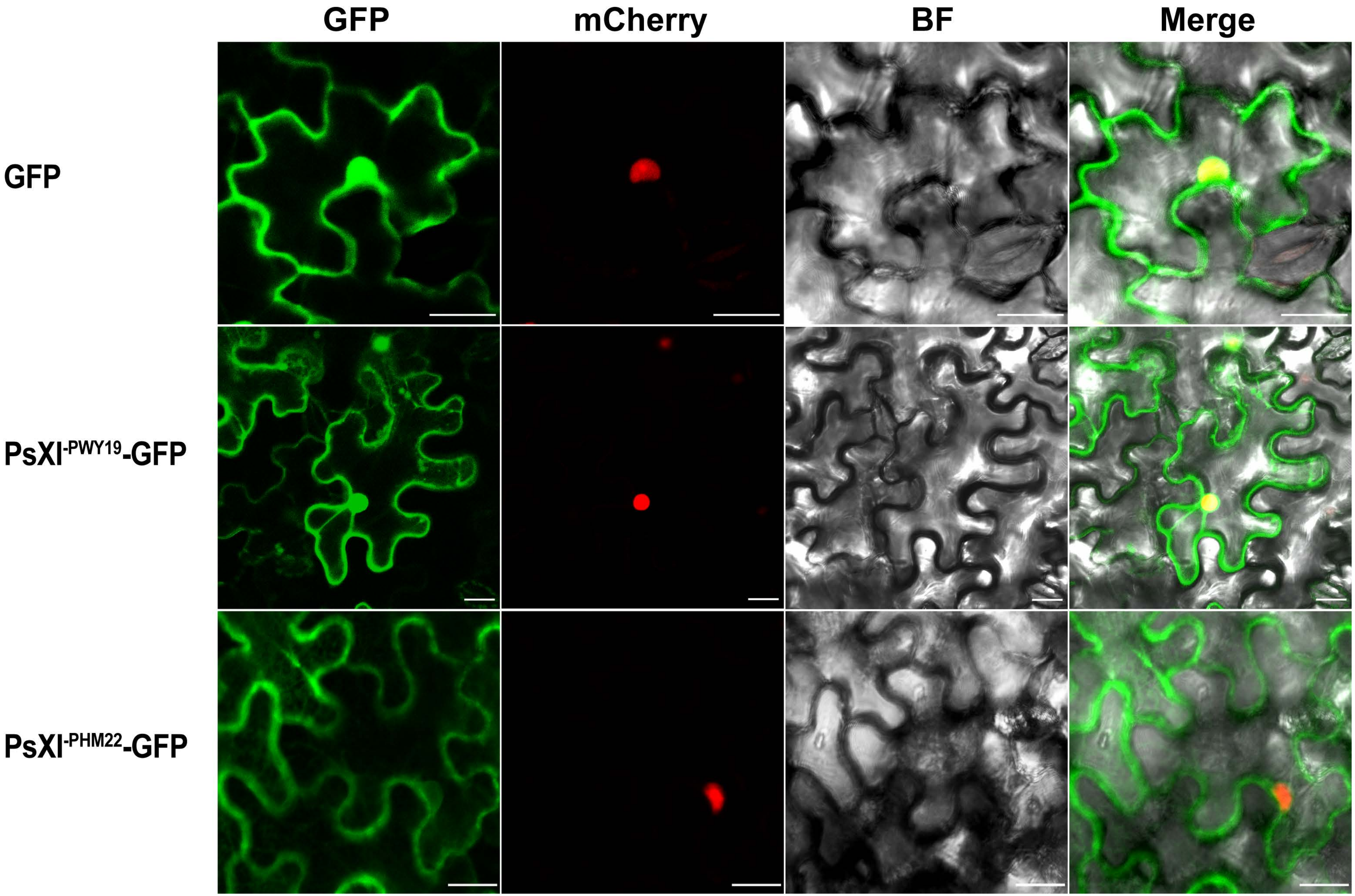

Supplementary Figure S6. S ubcellular localization of PsXI-GFP fusion proteins. GFP was used as the control. mCherry channel shows the marker of nucleus. BF, blight field. Scale bars, 20 μm
